# In Vivo Classification of Patellar Motion Trajectories in Individuals: A 4D-CT-Based Study with Unsupervised Clustering

**DOI:** 10.3390/diagnostics16101517

**Published:** 2026-05-16

**Authors:** Jiaying Wei, Ziyi Jiang, Xinhao Zhang, Weigen Ye, Bowen Guo, Weilin Wu, Jia Li, Mao Yuan, Dehua Wang, Hong Cheng, Wei Huang, Chen Zhao, Ke Li

**Affiliations:** 1 Orthopaedic Research Laboratory of Chongqing Medical University, Chongqing Municipal Health Commission Key Laboratory of Musculoskeletal Regeneration and Translational Medicine, Department of Orthopedics, The First Affiliated Hospital of Chongqing Medical University, Chongqing 400016, China; weijy0929@163.com (J.W.); 17853591682@163.com (D.W.); huangwei68@263.net (W.H.); 2 School of Automation Engineering, University of Electronic Science and Technology of China, Chengdu 611731, China; 202411060937@std.uestc.edu.cn (Z.J.); xhzhang_uestc@163.com (X.Z.); 2022190501013@std.uestc.edu.cn (W.Y.); 2024120101008@std.uestc.edu.cn (B.G.); wuweilin31@gmail.com (W.W.); hcheng@uestc.edu.cn (H.C.); 3 Department of Radiology, The First Affiliated Hospital of Chongqing Medical University, Chongqing 400016, China; 2h3864@hospital.cqmu.edu.cn (J.L.); miayuan113@163.com (M.Y.)

**Keywords:** four-dimensional computed tomography, artificial intelligence, patellar motion trajectories, patellofemoral joint, unsupervised clustering

## Abstract

**Background:** Patellar motion trajectory (PMT) is a key kinematic parameter for evaluating patellofemoral joint (PFJ) stability, but traditional static imaging indices are unable to capture the dynamic six-degrees-of-freedom (6-DOF) characteristics of patellar motion throughout the entire knee flexion–extension cycle. Four-dimensional computed tomography (4D-CT) facilitates in vivo dynamic imaging of the PFJ, while the systematic classification of PMT in asymptomatic populations has remained underexplored. **Methods:** A retrospective cross-sectional study was performed on 64 asymptomatic and functionally normal knees that underwent 4D-CT dynamic scanning from March 2021 to December 2025. Patellar 6-DOF kinematic data during 0° to 90° of knee flexion–extension were extracted through manifold optimization, automatic segmentation, and spatial registration. Following standardization of the motion cycle, unsupervised K-means clustering was employed to classify PMT phenotypes, with nonparametric tests used to analyze intergroup kinematic differences and evaluate clustering quality. **Results:** Three distinct PMT types were identified based on clustering validity indices, including a silhouette score of 0.381, a Davies-Bouldin index of 0.916, and a Calinski–Harabasz index of 44.06: Type 1 (7.81%, 35.11 ± 6.56 mm), Type 2 (56.25%, 15.67 ± 6.59 mm), and Type 3 (35.94%, 2.82 ± 2.41 mm). Lateral translation (Tx) served as the dominant determinant for PMT typing (*p* < 0.001), whereas non-lateral DOF parameters exhibited no consistent intergroup differences. Postural DOFs exhibited coupled fluctuations with Tx but had no independent stratification effect. Traditional static imaging parameters demonstrated no consistent correlation with these dynamic subtypes. **Conclusions:** Functionally asymptomatic knees exhibited three in vivo patellar 6-DOF motion trajectory phenotypes dominated by lateral translation amplitude. This 4D-CT-based typing framework provides a dynamic kinematic baseline for PFJ stability evaluation and lays a foundation for individualized optimization of ligament reconstruction and pathophysiological research of patellofemoral disorders.

## 1. Introduction

The patellofemoral joint (PFJ) functions as a critical functional unit of the knee, responsible for transmitting power from the extensor mechanism and redistributing joint loads during locomotion [1]. Patellar motion trajectory (PMT), which describes the continuous spatial displacement of the patella relative to the femur throughout the full knee flexion–extension cycle, encompasses six-degrees-of-freedom (6-DOF) kinematic features [2]. Biomechanically, a stable and coordinated PMT is essential for maintaining balanced patellofemoral contact stress, thereby ensuring that articular cartilage remains within a physiological load-bearing range [3]. Conversely, abnormal PMT can disrupt contact pressure distribution and induce excessive shear stress, thereby increasing the risk of patellofemoral pathologies, such as patellofemoral pain syndrome and recurrent patellar dislocation [4]. Thus, PMT is recognized as a key kinematic parameter for evaluating dynamic joint stability and mechanical coordination, as well as a fundamental basis for elucidating the pathophysiological mechanisms underlying patellofemoral dysfunction [5,6].

Despite its clinical significance, PMT assessment has long been reliant on indices derived from static imaging, including the Insall–Salvati index, congruence angle, trochlear groove angle, and tibial tuberosity–trochlear groove (TT-TG) distance [7,8,9]. While these parameters provide value for preliminary screening, their inherent limitations limit the comprehensive characterization of patellar kinematics. Specifically, measurements are typically obtained at fixed body positions or single flexion angles, thus failing to capture the continuous dynamic changes that occur during active knee motion [10,11]. Additionally, the manual measurement of these indices is prone to inter-observer variability. For example, the TT-TG distance can vary by up to 3 mm depending on whether osseous or cartilaginous landmarks are used, and anatomical landmark identification may vary among researchers [12]. Furthermore, static parameters cannot capture transient displacement fluctuations during continuous flexion–extension, and existing attempts at qualitative PMT characterization are limited by small sample sizes, insufficient analytical rigor, and a lack of systematic quantitative data across the complete motion cycle [13,14,15,16]. Collectively, the reliance on static indices leads to an incomplete understanding of the three-dimensional dynamic kinematic characteristics of the patella.

Recent advances in dynamic volumetric imaging have mitigated some of these limitations, with four-dimensional computed tomography (4D-CT) emerging as a powerful tool for joint kinematics research [17,18]. Unlike traditional single-phase 2D/3D imaging, 4D-CT acquires time-resolved three-dimensional volumetric data during continuous motion scanning, thereby enabling the comprehensive recording of knee joint spatial changes throughout the full flexion–extension cycle. This multi-phase data structure provides continuous input for 6-DOF motion analysis, thereby making feasible the reconstruction of trajectories across the complete motion cycle. However, the complexity of multi-phase volumetric data poses significant challenges for data processing, as bony structures across different time frames require precise segmentation, registration, and unification of coordinate systems to enable inter-phase comparisons. Fortunately, recent advancements in artificial intelligence (AI)-based algorithms have enhanced the automation of medical image segmentation and anatomical landmark recognition, with high repeatability and consistency demonstrated in bony structure segmentation and spatial localization [19,20,21]. Integration of these automated processing methods into kinematic modeling workflows enhances data-processing stability and, thereby, provides a reliable technical foundation for continuous trajectory analysis.

Although these technological advancements have been made, systematic identification of structurally meaningful motion patterns within complex three-dimensional time series remains insufficiently explored. Existing studies primarily focus on individual subjects or isolated DOF indices, with a paucity of research investigating the typing structure of multi-DOF coupled motion during the complete flexion–extension cycle. To address this gap, the present study utilized 4D-CT to acquire in vivo, full-range three-dimensional dynamic data, combined with automatic segmentation and spatial registration methods, to perform continuous modeling and 6-DOF kinematic analysis of patellar motion in functionally asymptomatic individuals under a unified coordinate system. The primary objectives were to identify reproducible PMT phenotypes and their key dynamic parameters, construct a kinematic typing framework under physiological conditions, and provide a kinematic basis for subsequent research on the optimization of ligament reconstruction parameters.

## 2. Materials and Methods

### 2.1. Study Population

This retrospective cross-sectional study collected and analyzed the imaging data of subjects who underwent dynamic knee joint 4D-CT scanning at The First Affiliated Hospital of Chongqing Medical University between March 2021 and December 2025. This study was approved by the Ethics Committee of The First Affiliated Hospital of Chongqing Medical University (Approval No. 2021-105), and all subjects signed informed consent forms. The study was registered with the Chinese Clinical Trial Registry (ChiCTR2200063223). To establish a baseline trajectory model, asymptomatic knees without patellofemoral joint dysfunction were strictly selected.

The inclusion criteria were (1) age between 18 and 50 years with mature skeletal development; (2) no previous knee joint surgery history, fracture history, or trauma history; and (3) negative clinical physical examination, including but not limited to no positive patellar apprehension test, no patellar instability, and normal physical evaluation of patellar trajectory.

The exclusion criteria are (1) imaging suggesting combined meniscal tear or injury of cruciate ligament and collateral ligament; (2) obvious patellofemoral anatomical abnormalities to exclude potential kinematic deviations, including but not limited to trochlear dysplasia (Dejour classification A-D type, or proximal trochlear groove angle ≥ 150°), patella alta (Insall–Salvati index ≥ 1.2) and excessive lateral patellar tilt (patellar tilt angle ≥ 16°); (3) presence of moderate to advanced knee degenerative changes or traumatic arthritis; and (4) inability to cooperate with the requirements of 4D-CT dynamic flexion–extension scanning due to pain, fear, or muscle spasm.

### 2.2. 4D-CT Dynamic Scanning for Raw Data Acquisition

All 4D-CT scans were performed by a single board-certified radiologist using a Canon Medical Systems 320-detector row Aquilion ONE dynamic volumetric CT system (Aquilion ONE, Canon Medical Systems, Otawara, Japan), eliminating interobserver variability and ensuring measurement consistency. Subjects were placed in a standardized supine position, with the proximal thigh immobilized by a customized brace and the calf freely mobile for unobstructed natural knee flexion–extension during scanning. Lead shielding was applied to the thyroid and gonads, in compliance with the as low as reasonably achievable principle for clinical radiation protection. Fixed scanning parameters were set as tube voltage 100 kV, tube current 70 mA, 512 × 512 acquisition matrix, 0.5 mm collimation and slice thickness, 0.35 s gantry rotation time, and 150 mm × 150 mm field of view focused on the scanned knee. Ten-second dynamic scans captured continuous knee motion from full extension at approximately 0° to maximum flexion at approximately 90°, acquiring 20–30 high-fidelity 3D volumetric datasets per flexion-extension cycle to resolve patellar spatiotemporal kinematics. A senior radiologist monitored all scans in real time, controlling the flexion–extension frequency to minimize motion-induced step artifacts. Post-acquisition, two senior physicians independently reviewed all raw tomographic images. Datasets with an inadequate signal-to-noise ratio or severe motion blur were excluded to ensure uniform slice thickness and anatomical detail across included data, providing a robust foundation for subsequent high-precision modeling and AI-based trajectory extraction.

### 2.3. Experimental Methods

#### 2.3.1. Reconstruction of Patellofemoral Joint 3D Model and Extraction of Motion Trajectory

**Figure 1:**
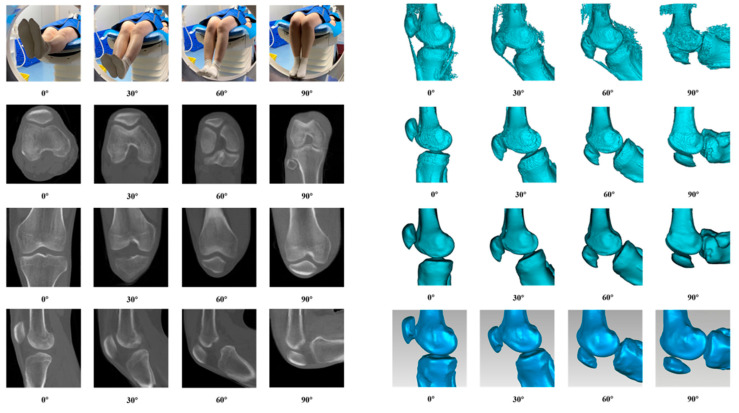
Schematic illustration of in vivo patellar tracking and 3D reconstruction during active knee flexion.

Raw 4D-CT IMA data were converted to a standard DICOM format via Python (v3.13.0) in Visual Studio Code (v1.94.2), followed by standardized preprocessing and spatial cropping to remove non-target anatomical structures. Preprocessed sequences were imported into Mimics Medical (v26.0, Materialise, Belgium) for segmentation. Femoral and patellar structures were isolated via adaptive Hounsfield unit thresholding, with manual correction by orthopedic surgeons to ensure bony boundary accuracy, yielding 0.5 mm voxel resolution knee models. All flexion-angle models were refined in Geomagic Studio (v2023, 3D Systems, USA) software, with non-manifold edge removal, micro-defect repair, and bilateral filtering for surface smoothing. Closed patellofemoral joint models in STL and PLY formats were generated via non-uniform rational B-splines surface reconstruction, with irrelevant tibial and fibular structures excluded. A four-dimensional time–series motion model was constructed for continuous kinematic quantification. The anatomically stable femur was set as the global spatial reference, with iterative closest-point registration unifying all flexion-angle bone models into a single coordinate system. Patellar spatial centroid coordinates at each discrete phase were extracted via automated scripts, with nonlinear curve fitting enabling accurate characterization and digital reconstruction of six-degree-of-freedom patellar motion paths across the full knee flexion–extension range. The process is shown in Figure 1.

#### 2.3.2. Definition of Anatomical Reference Landmarks and Coordinate System

The native coordinate system embedded in the point cloud data of the 3D patellofemoral joint model was designated as the world coordinate system, and a femur-based local coordinate system was established. The midpoint of the medial and lateral femoral condyles was set as the origin of the local coordinate system. The line connecting the medial and lateral femoral condyles was defined as the X-axis, with the positive direction oriented from medial to lateral. The XOY plane was constructed using the two femoral condyles and the femoral anatomical axis. The normal vector of the XOY plane passing through the origin was defined as the Z-axis, with the positive direction oriented from distal to proximal. The Y-axis was subsequently determined via the right-hand rule. This coordinate system corresponded to the medial–lateral, anterior–posterior, and proximal–distal anatomical directions to the X, Y, and Z axes, respectively, providing a unified mathematical framework for subsequent spatial kinematic analysis.

#### 2.3.3. Unsupervised 4D Trajectory Reconstruction and Typing Based on Manifold Optimization

We developed an automated 4D-CT-based computational framework to extract high-fidelity patellofemoral kinematic features, comprising four core steps: standardized image preprocessing, manifold optimization-driven 3D kinematic reconstruction, biphasic spatiotemporal normalization, and unsupervised subspace clustering for trajectory phenotyping.

##### Four-Dimensional Kinematic Trajectory Reconstruction via Manifold Optimization

Let $P=\left\{P_{k}|k=0,1,\ldots ,N-1\right\}$ denote patellar surface point clouds reconstructed from N dynamic CT frames, where $P_{k}={\left\{p_{k,j}\in R^{3}\right\}}_{j=1}^{M_{k}}$ is the k-th frame point set. Geometric centering was first performed to mitigate lever-arm effect-induced numerical instability, as the patella lies far from the native CT coordinate origin. The centroid of the 0-th frame c_0_ was set as the reference origin, with a translation transform applied to each frame’s point cloud (1). This transform decoupled motion into pure rotational and translational components relative to the anatomical reference origin. To estimate the 6-DOF pose of the k-th frame relative to the initial reference frame k = 0, we solved for the rigid-body transform $T_{k}\in SE\left(3\right)$ via a combined inter-frame and template registration strategy to preserve trajectory continuity and robustness. With the relative transform defined as $T_{k-1,k}=T_{k-1}^{-1}T_{k}$, the pose optimization problem was formulated as a nonlinear least-squares problem minimizing point-to-surface error, with a Lie algebra $se\left(3\right)$-based regularization constraint to mitigate cumulative error and registration drift. Let $\xi_{k}\in R^{6}$ denote the Lie algebra coordinates corresponding to $T_{k}$. The optimization objective function was defined as (2), where $r_{\mathrm{odom}}$ denotes the odometry residual between adjacent frames, $r_{\mathrm{map}}$ denotes the matching residual between the current frame and first-frame template, S denotes the set of high-confidence template-matching frames, and λ is the Lagrange multiplier for weight balancing. The residual term was defined in the tangent space of $se\left(3\right)$ as (3). This problem was solved via the Levenberg–Marquardt algorithm with a Huber kernel. Samples with optimized translation amplitude $∥t_{k}∥>\tau_{t}$ or goodness of fit $\epsilon <\tau_{f}$ were excluded to ensure kinematic estimate plausibility.

(1) $$\;{\tilde{P}}_{k}=\left\{p-c_{0}|p\in P_{k}\right\}$$

(2) $$\underset{\left\{\xi_{k}\right\}}{\min}\sum_{k=1}^{N-1}\left(∥r_{\mathrm{odom}}\left(T_{k-1},T_{k}\right){∥}_{\Sigma_{o}}^{2}+\lambda I\left(k\in S\right)∥r_{\mathrm{map}}\left(T_{0},T_{k}\right){∥}_{\Sigma_{m}}^{2}\right)$$

(3) $$\;r_{i,j}=\mathrm{Log}\left(T_{i}^{-1}T_{j}\right)\in se\left(3\right)$$

##### Spatiotemporal Standardization of Kinematic Data

To enable valid cross-subject statistical comparisons, we eliminated confounding effects from left–right knee asymmetry and variable motion cycle duration. All patellar models were first registered to a unified femoral anatomical coordinate system via the iterative closest point algorithm. For left knee samples, symmetric mirror mapping $M:R^{6}\to R^{6}$ was applied to unify kinematic semantics, with the 6-DOF trajectory vector defined as $x_{k}={\left[Tx,Ty,Tz,Rx,Ry,Rz\right]}_{k}^{T}$ (4). To preserve the asymmetric hysteresis of knee flexion-extension dynamics lost with standard single-phase linear interpolation, we developed a keyframe-based biphasic resampling method. The peak flexion frame index $k_{\mathrm{peak}}$ was identified from longitudinal displacement Tz (5). The full motion cycle was split into flexion and extension phases, with a uniform 25-point grid constructed for each phase. The standardized feature vector was generated as (6), where $\Psi_{\mathrm{interp}}$ denotes an extremum-preserving linear interpolation operator. A second-order 7-point Savitzky–Golay filter was applied to suppress high-frequency quantization noise while preserving J-sign trajectory geometric features.

(4) $$\;{x^{\prime }}_{k}=\{\begin{array}{ll} {\left[-Tx,Ty,Tz,Rx,-Ry,-Rz\right]}^{T}, & \mathrm{if}\;\mathrm{case}\in \Omega_{L}\\ x_{k}, & \mathrm{otherwise} \end{array}$$

(5) $$\;k_{\mathrm{peak}}=\arg\underset{k\in \left[0,N-1\right]}{\max}\left|Tz_{k}\right|$$

(6) $$\;y={\left[\Psi_{\mathrm{interp}}\left(\left\{0\ldots k_{\mathrm{peak}}\right\},M\right),\;\Psi_{\mathrm{interp}}\left(\left\{k_{\mathrm{peak}}\ldots N-1\right\},M\right)\right]}^{T}\;$$

##### Unsupervised Clustering for Trajectory Phenotyping

We constructed an unsupervised subspace clustering model based on lateral displacement features to identify intrinsic patellar motion patterns without prior labeling. Principal component analysis was performed for feature space dimensionality reduction, mitigating confounding effects from variable knee flexion depth. The feature matrix $X\in R^{S\times 50}$ was projected into a low-dimensional subspace via an orthogonal basis $W\in R^{50\times d}$ (7), where d was selected to retain cumulative explained variance ≥ 95% (8). K-means clustering was performed in the reduced latent space z to identify optimal trajectory clusters, with the objective function minimizing the within-cluster sum of squared errors (9). The optimal cluster number K was determined via joint evaluation of the elbow method inflection point and silhouette coefficient. To endow clustering results with clinical and biomechanical interpretability, we defined the order parameter $\gamma_{j}$ as the mean maximum lateral displacement of the j-th cluster (10). Cluster labels were reordered according to $\gamma_{j}$ to generate an interpretable lateral-translation gradient, ranging from the greatest lateral displacement phenotype to the most stable patellar tracking phenotype.

(7) $$\;Z=\left(X-\mu_{X}\right)W$$

(8) $$\sum_{i=1}^{d}\lambda_{i}/\sum \lambda_{total}\geq 95\%$$

(9) $$\underset{\left\{c_{j}\right\},\left\{z_{i}\right\}}{\min}\sum_{j=1}^{K}\sum_{z_{i}\in C_{j}}∥z_{i}-c_{j}{∥}^{2}$$

(10) $$\gamma_{j}=\frac{1}{\left|C_{j}\right|}\sum_{i\in C_{j}}\max\left(v_{i}\right)$$

### 2.4. Statistical Analysis

Normality was assessed using the Shapiro–Wilk test. Because several variables deviated from normality and the sample sizes of the trajectory phenotypes were unbalanced, a nonparametric statistical framework was used. The Kruskal–Wallis H test was applied for overall comparisons among the three phenotypes. For post hoc pairwise comparisons, the Brunner–Munzel test was used because it is robust to unequal sample sizes and heteroscedasticity. The *p* values were adjusted for multiple comparisons using the Benjamini–Hochberg false discovery rate procedure. A two-sided adjusted *p* < 0.05 was considered statistically significant. Rank–biserial correlation was reported as the effect size, with absolute values closer to 1 indicating stronger between-group separation. Additional sensitivity analyses were performed using Welch’s *t*-test, Student’s *t*-test, the Mann–Whitney U test, Kolmogorov–Smirnov test, Mood’s median test, and Anderson–Darling test.

## 3. Results

### 3.1. Typing Results and Overall Characteristics of In Vivo Patellar Motion Trajectories

**Figure 2:**
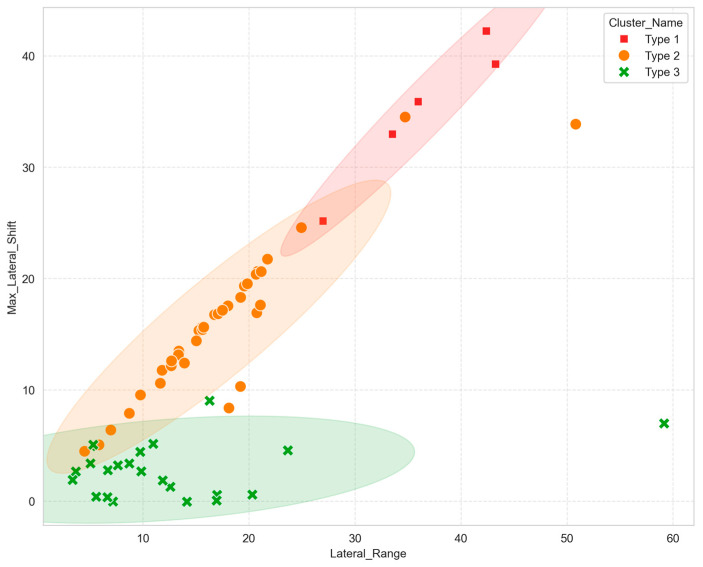
Scatter plot of the three identified patellar motion trajectory phenotypes according to core lateral kinematic parameters. The x-axis indicates lateral range of motion, and the y-axis indicates maximum lateral patellar shift. Type 1, Type 2, and Type 3 are shown with different markers. The distribution demonstrates a clear lateral-translation gradient among the three phenotypes, although partial overlap between adjacent phenotypes may exist because patellar tracking represents a continuous biological spectrum.

A total of 64 knee joint samples were included in the final analysis, with detailed baseline demographic characteristics, patellar and femoral morphological parameters, and static imaging measurements presented in Table 1. All included knees were asymptomatic and had no clinically diagnosed patellofemoral dysfunction according to the predefined inclusion and exclusion criteria. After manifold optimization-driven in vivo 4D patellar motion trajectory reconstruction, we used unsupervised K-means clustering to categorize 6-DOF kinematic features across the standardized flexion–extension cycle. A three-cluster solution was selected based on the elbow method and clustering validity indices, including a silhouette coefficient of 0.381, Davies–Bouldin index of 0.916, and Calinski–Harabasz index of 44.06. We identified three distinct in vivo patellar motion trajectory phenotypes with unique kinematic and spatial features, designated Type 1 (marked in red), Type 2 (marked in orange), and Type 3 (marked in green). Type 1 included five samples, accounting for 7.81% of the cohort. Type 2 included 36 samples, accounting for 56.25% of the cohort, and Type 3 included 23 samples, accounting for 35.94% of the cohort. Type 2 was the predominant phenotype, comprising over half of the total cohort, followed by Type 3 (Figure 2). The silhouette coefficient indicated moderate cluster separation rather than complete isolation, which is expected for continuous biological kinematic data. However, the combined results of the elbow method, Davies–Bouldin index, Calinski–Harabasz index, and the stepwise gradient of lateral translation supported a three-cluster solution with clear biomechanical interpretability. Partial overlap between adjacent phenotypes, particularly between Type 2 and Type 3, may reflect the continuous nature of patellar motion rather than a strict categorical boundary.

### 3.2. Spatial Morphological Features of Trajectory Phenotypes

**Figure 3:**
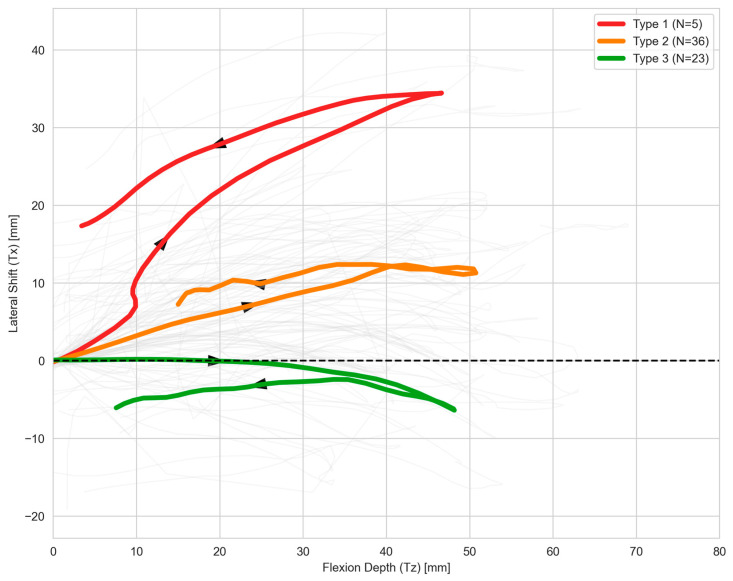
Mean spatial motion loops of three patellar motion trajectory phenotypes during the knee flexion–extension cycle. The x-axis represents Tz displacement (mm), and the y-axis represents lateral patellar shift (Tx, mm). Type 1 is shown in red, Type 2 in orange and Type 3 in green. Black arrows indicate the direction of knee flexion. The black dashed line marks the zero lateral displacement baseline, and faint gray lines display individual raw patellar trajectories. A clear gradient of lateral patellar excursion is observed across the three phenotypes.

The three patellar trajectory phenotypes exhibited distinct spatial morphological characteristics on lateral projection across the standardized flexion–extension cycle, with these characteristics showing high consistency with our prior quantitative kinematic findings. Type 1 exhibited a markedly exaggerated lateral displacement pattern throughout the flexion–extension cycle, with its spatial path forming a highly asymmetric curved loop structure consistent with a prominent patellofemoral S-sign morphology. The Type 1 trajectory was globally displaced from the midline of the femoral trochlear groove, with significantly increased lateral excursion across the full motion cycle. Type 2 presented a moderate lateral displacement phenotype, with a demonstrable J-sign in its spatial path. Its lateral displacement amplitude and loop curvature were significantly reduced relative to Type 1, forming a transitional L-sign-like morphology. Type 3 exhibited a gently curved spatial path with a typical C-sign morphology, with minimal lateral excursion and a motion path closely aligned with the femoral trochlear groove midline, representing a relatively stable patellar tracking pattern (Figure 3).

### 3.3. Kinematic Differences Across Trajectory Phenotypes

To quantitatively characterize the kinematic differences between patellar trajectory phenotypes, we analyzed two lateral-translation metrics, maximum lateral displacement and lateral range of motion, together with maximum longitudinal displacement (Tz) as a reference non-lateral metric reflecting motion depth. Shapiro–Wilk testing confirmed significant normality deviations for multiple metrics across groups, so all pairwise between-group comparisons were performed using the nonparametric Brunner–Munzel test with Benjamini–Hochberg false discovery rate (BH-FDR) correction. Rank–biserial correlation was reported as the effect size, with absolute values closer to one indicating greater between-group differences. Full descriptive statistics and post hoc pairwise comparisons for the three core kinematic variables are summarized in Table 2. To improve interpretability, each adjusted *p* value and effect size is reported together with its corresponding pairwise comparison.

Maximum lateral displacement exhibited a clear stepwise gradient ranked Type 1 > Type 2 > Type 3, with highly significant differences in all pairwise comparisons and all adjusted *p* values < 0.001. Type 1 had the highest maximum lateral displacement at 35.11 ± 6.56 mm, median 35.90 mm, more than twice that of Type 2 at 15.67 ± 6.59 mm, median 15.50 mm, and approximately 12-fold higher than Type 3 at 2.82 ± 2.41 mm, median 2.66 mm. The over 30 mm mean difference between Type 1 and Type 3 corresponded to an effect size of −1.000, indicating near-complete phenotype separation for this metric.

**Figure 4:**
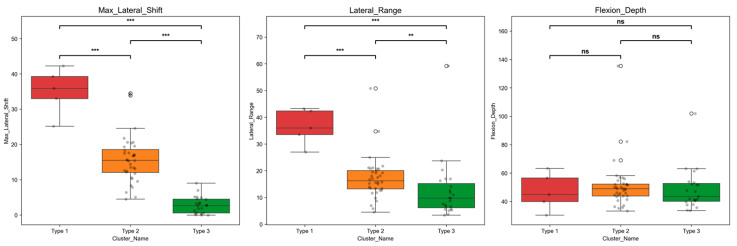
Box-and-whisker plots of key kinematic parameters across three patellar motion trajectory phenotypes. Box plots display the median, interquartile range, and range of maximum lateral shift (**left**), lateral range of motion (**middle**), and maximum longitudinal displacement (**right**) for Type 1 (red), Type 2 (orange), and Type 3 (green) phenotypes. Pairwise between-group comparisons were performed using the Brunner–Munzel test with BH-FDR correction. Significance markers: *** *p* < 0.001; ** *p* < 0.01; ns = no statistically significant difference.

Lateral range of motion followed the same stepwise gradient, with values of 36.42 ± 6.71 mm in Type 1, 17.17 ± 8.11 mm in Type 2, and 12.53 ± 11.55 mm in Type 3. All pairwise comparisons were statistically significant: Type 1 versus Type 2 had an adjusted *p* value < 0.001 and RBC = −0.922; Type 1 versus Type 3 had an adjusted *p* value < 0.001 and RBC = −0.913; and Type 2 versus Type 3 had an adjusted *p* value = 0.002 and RBC = −0.493. Time–series analysis of lateral translation Tx showed that these differences stemmed from persistent exaggerated lateral displacement across the full flexion–extension cycle, not transient single-angle abnormalities. Type 1 showed rapid early-flexion displacement and sustained high excursion through mid-flexion, remaining consistently elevated across the cycle. Type 2 had moderate displacement. Type 3 showed minimal fluctuation with a near-flat translation curve. In contrast to the lateral kinematic metrics, maximum longitudinal displacement showed no significant between-group differences across all pairwise comparisons, with all adjusted *p* values > 0.05 and small effect sizes. The mean maximum longitudinal displacement was 46.97 ± 13.13 mm for Type 1, 51.09 ± 17.19 mm for Type 2, and 48.46 ± 14.67 mm for Type 3, with highly overlapping group distributions. These findings confirm comparable longitudinal displacement-related motion across all phenotypes, which did not drive the observed between-phenotype differences in patellar motion trajectories (Figure 4).

### 3.4. 6-DOF Kinematic Evolution Patterns of Patellar Trajectories

We further analyzed the dynamic evolution of all six-degrees-of-freedom parameters across the standardized flexion-extension cycle to identify core drivers of patellar trajectory phenotyping. Descriptive statistics and post hoc pairwise comparisons for the 6-DOF kinematic parameters are presented in Table 3, and the structural interpretation of each degree of freedom is summarized in Table 4.

Lateral translation Tx was the dominant determinant of trajectory phenotyping, forming the most stable hierarchical structure across the three phenotypes. The Tx peak showed large-to-extreme absolute RBC values ranging from 0.956 to 1.000, while the total range of Tx showed absolute RBC values ranging from 0.493 to 0.922, with continuous time–series curve separation across the full flexion cycle.

The posture-related parameters, including patellar tilt Rx and axial rotation Rz, showed consistent cyclic fluctuations across all phenotypes with largely overlapping time–series curves. Type 1 exhibited synchronous Rx and Rz fluctuations during peak lateral displacement, with moderate effect sizes in some pairwise comparisons reaching a maximum RBC = 0.530 for Rx and 0.556 for Rz. These differences did not survive BH-FDR correction, with all adjusted *p* > 0.05, and failed to form a stable inter-phenotype hierarchical structure. These findings indicate Rx and Rz are likely secondary coordinated adjustments to exaggerated lateral translation, not core drivers of trajectory phenotyping.

**Figure 5:**
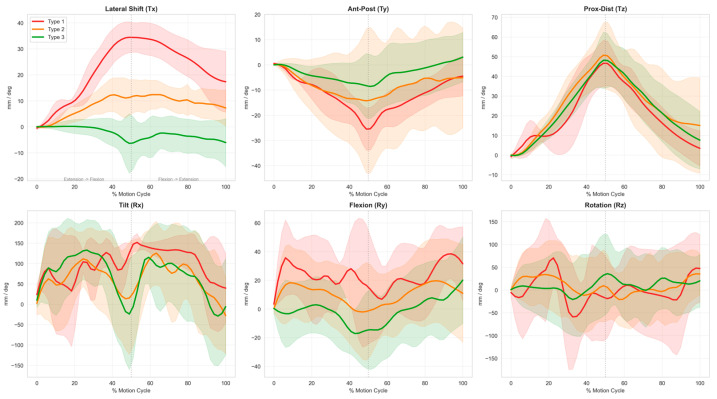
Average 6-DOF kinematic curves across the standardized knee flexion–extension cycle. Solid lines represent the mean value for each phenotype, with shaded areas indicating the standard deviation. The x-axis denotes the normalized knee motion cycle, and the y-axis indicates displacement (mm) for translational degrees of freedom (lateral shift Tx, anterior–posterior translation Ty, proximal–distal translation Tz) and rotation angle (°) for rotational degrees of freedom (tilt Rx, flexion Ry, axial rotation Rz). Type 1 is shown in red, Type 2 in orange, and Type 3 in green. A consistent stepwise gradient of Tx is observed across the three phenotypes throughout the full motion cycle.

The remaining three parameters, anterior–posterior translation Ty, proximal–distal translation Tz, and patellar flexion Ry, showed highly consistent dynamic trends across phenotypes, with extensively overlapping time–series curves. No significant differences were observed in peak amplitude or fluctuation range for Tz and Ry across any pairwise comparisons, with all adjusted *p* > 0.05 and small effect sizes with |RBC| < 0.3. A single significant difference in maximum Ty displacement was observed between Type 2 and Type 3, with an adjusted *p* value = 0.019 and RBC = −0.425; however, this did not form a systematic three-phenotype gradient, and no differences were found in total Ty range of motion. Collectively, these parameters reflect secondary or baseline kinematic features of global knee flexion–extension and do not contribute substantially to structural differences between trajectory phenotypes (Figure 5).

### 3.5. Comparative Analysis Between Dynamic PMT Phenotypes and Traditional Static Imaging Parameters

**Figure 6:**
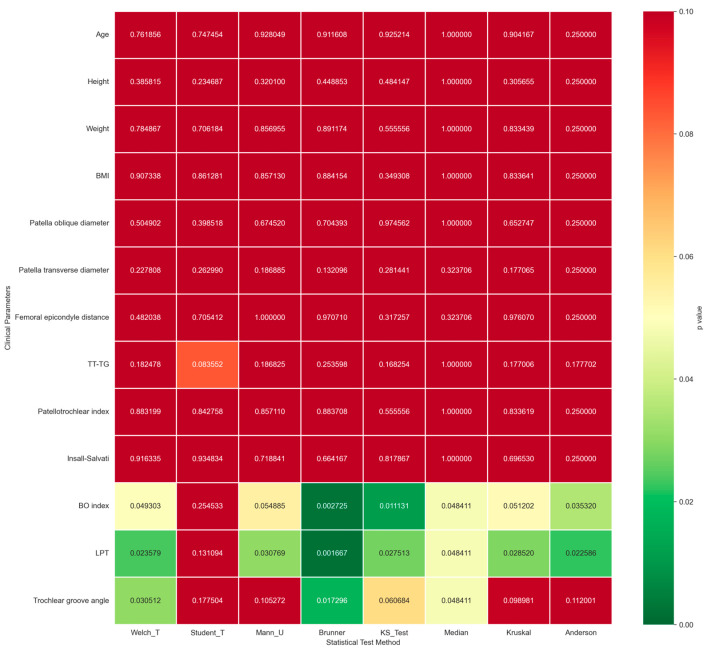
Heatmap of *p* values for between-group comparisons of static clinical and imaging parameters. Rows represent demographic and static patellofemoral imaging parameters, and columns represent 8 distinct statistical test methods. The color gradient indicates the magnitude of *p* values, with darker green corresponding to smaller *p* values and darker red corresponding to larger *p* values. No static parameter showed a consistently significant between-group difference across all statistical tests, confirming the absence of a stable association between individual static structural variables and dynamic patellar trajectory phenotypes.

We performed a robustness analysis to assess the associations between our previously identified dynamic patellar trajectory phenotypes and traditional static clinical and imaging parameters. The Brunner–Munzel test, which is optimized for settings with unbalanced sample sizes and heteroscedasticity, was used as the primary analytical approach, with complementary robustness validation performed using seven alternative statistical methods: Welch’s *t*-test, Student’s *t*-test, the Mann–Whitney U test, Kruskal–Wallis H test, Kolmogorov–Smirnov test, Mood’s median test, and the Anderson–Darling test. Demographic parameters, including age, height, weight, and body mass index, showed no significant between-group differences across phenotype groups in the primary Brunner–Munzel test, with all *p* values > 0.05. The small observed effect sizes confirmed no systematic confounding effect of baseline demographic factors on trajectory phenotyping. Static imaging parameters, including the Insall–Salvati index, trochlear groove angle, and other osseous morphological metrics, similarly showed no consistent significant between-group differences in the primary analysis. While individual parameters, including the bisect offset (BO) index with a *p* value of 0.0027 and lateral patellar tilt (LPT) with a *p* value of 0.0016, showed nominal statistical significance in the primary Brunner–Munzel test, these findings were not consistent across alternative statistical methods, and neither parameter retained statistical significance across all validation analyses (Figure 6). No static parameter demonstrated a directionally consistent and statistically significant association with dynamic patellar phenotypes across all statistical tests. This finding confirms that no stable one-to-one mapping exists between dynamic trajectory phenotypes and individual static structural variables.

## 4. Discussion

In this study, we reconstructed in vivo 6-DOF patellar kinematic trajectories from dynamic 4D-CT scans in a cohort of functionally asymptomatic knees and identified three distinct patellar motion phenotypes via unsupervised K-means clustering. Our core finding is that Tx is the dominant driver of trajectory heterogeneity, exhibiting a clear stepwise gradient across the three phenotypes throughout the full knee flexion–extension cycle. Critically, we identified no stable one-to-one mapping between traditional static clinical and imaging parameters and dynamic trajectory phenotypes, even for widely used clinical metrics, including the Insall–Salvati index and trochlear groove angle.

Current clinical risk stratification for patellofemoral joint dysfunction and adverse knee surgical outcomes relies largely on static anatomical risk factors, which are broadly categorized into osseous and soft tissue abnormalities, encompassing both static structural derangements and their downstream dynamic functional consequences [22,23]. The five core anatomical risk factors for patellar instability and maltracking are well-established: femoral trochlear dysplasia, patella alta, lateralization of the tibial tuberosity, excessive lateral patellar tilt, and femoral and/or tibial torsional malalignment. Additional recognized risk factors include knee valgus malalignment, patellar dysplasia, vastus medialis obliquus atrophy, generalized joint laxity, medial patellar retinaculum insufficiency, and lateral retinaculum contracture [24,25,26,27]. However, these static parameters cannot fully capture the in vivo dynamic kinematic behavior of the patellofemoral joint during active knee motion, which is the core mechanistic driver of patellar maltracking, secondary joint degeneration, and suboptimal functional recovery following knee surgery [28]. Our findings directly address a critical, longstanding gap in the existing research: prior studies have repeatedly reported significant discrepancies between the severity of static anatomical derangements and clinical symptom burden, as well as highly variable postoperative outcomes in patients with near-identical static imaging features [29,30,31,32]. The dynamic trajectory phenotypes identified in this study provide a quantifiable, kinematic explanation for this widespread clinical heterogeneity. Importantly, the dynamic PMT phenotypes were not consistently explained by traditional static imaging parameters. This finding suggests that 4D-CT-derived trajectory phenotyping captures functional kinematic information that is not redundant with conventional anatomical measurements. Static indices such as the Insall–Salvati index, TT-TG distance, trochlear groove angle, and patellar tilt angle describe osseous alignment or morphology at fixed positions, whereas PMT phenotyping reflects continuous patellar behavior during active knee motion. Therefore, dynamic trajectory analysis may complement, rather than replace, conventional imaging evaluation in the assessment of patellofemoral stability.

Of particular clinical relevance, the Type 1 phenotype, characterized by markedly lateral patellar displacement throughout the flexion–extension cycle, represents a high-risk dynamic pattern even in the absence of static pathological anatomical abnormalities [33,34,35]. Notably, the Type 1 phenotype represents an asymptomatic but kinematically atypical trajectory identified in our asymptomatic cohort, and several mechanisms may explain why these individuals remained clinically asymptomatic despite marked lateral patellar displacement. First, sufficient neuromuscular compensation, particularly from the vastus medialis obliquus and dynamic quadriceps control, may maintain functional patellar stability during active motion. Second, young or structurally resilient joints may tolerate abnormal patellar tracking without immediate symptoms. Third, the absence of pain or instability at the time of imaging does not exclude a preclinical or compensatory biomechanical state. Therefore, the Type 1 phenotype may represent a functional kinematic biomarker that identifies individuals with potential susceptibility to future patellofemoral dysfunction, rather than a pathological phenotype per se. This finding may have clinical relevance for patellofemoral disorders and selected procedures in which patellar tracking is an important determinant of functional outcome, particularly medial patellofemoral ligament reconstruction and surgical decision-making for patellar instability. In these settings, unrecognized abnormal patellar dynamic trajectories may contribute to persistent patellofemoral symptoms or suboptimal functional recovery. Therefore, the phenotyping framework established in this study may help identify high-risk dynamic patterns and support individualized evaluation of patellofemoral stability, rather than relying solely on static anatomical measurements [36,37].

Several limitations of the present study warrant acknowledgment. First, this is a single-center study with a modest overall sample size, particularly for the Type 1 phenotype, and the generalizability of our findings requires prospective validation in large-scale, multi-center cohorts. Second, our cohort was restricted to asymptomatic knees without clinically diagnosed patellofemoral dysfunction, and we did not perform quantitative in vivo measurement and analysis of native knee ligament tension in the current study, which precludes further elucidation of the intrinsic mechanistic link between ligament biomechanical properties and the identified patellar dynamic trajectory phenotypes. Third, soft-tissue laxity and neuromuscular control were not directly assessed in this cohort. Generalized joint hypermobility, medial soft-tissue restraint insufficiency, vastus medialis obliquus dysfunction, and lateral retinacular tightness may influence dynamic patellar tracking and may partly explain the exaggerated lateral translation observed in the Type 1 phenotype. Because these clinical variables could not be reliably collected in the present study, they were not included in the current analysis. Future studies are, therefore, needed to validate these trajectory phenotypes in patient populations with knee ligament injuries, patellofemoral pain, or frank patellar instability; incorporate targeted measurement and analysis of in vivo physiological and pathological ligament tension, standardized clinical laxity assessments, and quantitative evaluation of peri-patellar muscle function to clarify the underlying biomechanical mechanisms of distinct patellar trajectory phenotypes; and correlate these dynamic phenotypes with objective clinical outcomes in patients with patellofemoral disorders and after patellofemoral ligament-related procedures [38,39,40,41].

## 5. Conclusions

In conclusion, this study establishes a robust dynamic phenotyping framework for in vivo patellar motion based on quantitative 4D-CT kinematic analysis and identifies lateral patellar translation as the core determinant of trajectory heterogeneity in functionally asymptomatic knees. The absence of a stable correlation between static anatomical parameters and dynamic kinematic phenotypes highlights the critical unmet clinical need to integrate dynamic functional assessment into the routine preoperative evaluation of knee pathologies. Our findings provide a fundamental kinematic basis for personalized risk stratification and surgical planning in patellofemoral disorders and selected knee procedures in which patellar tracking is clinically relevant, with the potential to optimize patient-specific treatment strategies and improve long-term clinical outcomes.

## Figures and Tables

**Table 1:** Demographic characteristics and basic patellofemoral imaging parameters of the included knee models.

	Values a
Age, y	30.13 [7.15]
Height, cm	169.03 [8.39]
Weight, kg	68.09 [13.74]
Sex	
Female	23 (35.94%)
Male	41 (64.06%)
Left and Right of Knee Joint Models	
Left	30 (46.88%)
Right	34 (53.13%)
Gelsamer Classification of the Patella	
I	44 (68.75%)
II	10 (15.63%)
III	10 (15.63%)
Wiberg Classification of the Patella	
I	11 (17.19%)
II	29 (45.31%)
III	24 (37.50%)
Patellar basic values	
Patella Oblique Diameter, mm	40.62 [4.03]
Patella Transverse Diameter, mm	44.99 [3.07]
Intercondylar Distance Between the Medial and Lateral Epicondyles of the Femur, mm	80.78 [5.76]
Patella Height	
Insall–Salvati Index	0.93 [0.09]
Patella Trochlear Index	0.69 [0.21]
Trochlear Groove Morphology	
Trochlear Groove Angle, Degrees	126.87 [8.73]
Axial Linear Displacement of the Patella	
Patella Bisecting-Offset (BO) Index	63.40 [11.05]
Axial Tilt of the Patella	
Patella Tilt Angle, Degrees	9.98 [4.65]
Lateralization of the Tibial Tuberosity	
Tibial Tuberosity–Trochlear Groove Distance, mm	9.96 [6.26]

**Note:** a. Normal reference ranges are provided for clinical interpretation: Insall–Salvati Index (0.8–1.2), Patella Trochlear Index (0.125–0.80), Trochlear Groove Angle (138° ± 6°), Patella Bisecting-Offset (BO) Index (44–66), Patella Tilt Angle (<12°), and tibial tuberosity–trochlear groove distance (<20 mm). Values are presented as mean [standard deviation] and number (percentage).

**Table 2:** Descriptive statistics and pairwise comparisons of representative kinematic variables across PMT phenotypes.

Variable	Phenotype	Mean ± SD	Median [IQR]	Pairwise Comparison	Adjusted *p* Value	RBC	Significance
Maximum Lateral displacement (mm)	Type 1	35.11 ± 6.56	35.90 [6.29]	T1 vs. T2	<0.001	−0.956	***
	Type 2	15.67 ± 6.59	15.50 [6.50]	T1 vs. T3	<0.001	−1.000	***
	Type 3	2.82 ± 2.41	2.66 [3.92]	T2 vs. T3	<0.001	−0.966	***
Lateral range of motion (mm)	Type 1	36.42 ± 6.71	35.95 [8.86]	T1 vs. T2	<0.001	−0.922	***
	Type 2	17.17 ± 8.11	16.25 [6.87]	T1 vs. T3	<0.001	−0.913	***
	Type 3	12.53 ± 11.55	9.75 [9.07]	T2 vs. T3	0.002	−0.493	**
Maximum longitudinal displacement (mm)	Type 1	46.97 ± 13.13	44.87 [16.63]	T1 vs. T2	0.954	0.111	ns
	Type 2	51.09 ± 17.19	48.95 [8.37]	T1 vs. T3	0.954	−0.026	ns
	Type 3	48.46 ± 14.67	43.53 [12.56]	T2 vs. T3	0.954	−0.152	ns

Note: Data are presented as mean ± standard deviation and median [interquartile range]. Type 1, Type 2, and Type 3 included 5, 36, and 23 knees, respectively. Pairwise comparisons were performed using the Brunner–Munzel test with Benjamini–Hochberg false discovery rate correction. RBC indicates rank–biserial correlation. Significance was defined by adjusted *p* values. *** *p* < 0.001; ** *p* < 0.01; ns, not significant.

**Table 3:** Descriptive statistics and between-group comparisons of 6-DOF patellar kinematic parameters across trajectory phenotypes.

Kinematic Parameter	Type 1	Type 2	Type 3	Pairwise Comparison	Adjusted *p* Value	RBC	Significance
Tx peak (mm)	35.11 ± 6.56	15.67 ± 6.59	2.82 ± 2.41	T1 vs. T2	<0.001	−0.956	***
				T1 vs. T3	<0.001	−1.000	***
				T2 vs. T3	<0.001	−0.966	***
Total range of Tx (mm)	36.42 ± 6.71	17.17 ± 8.11	12.53 ± 11.55	T1 vs. T2	<0.001	−0.922	***
				T1 vs. T3	<0.001	−0.913	***
				T2 vs. T3	0.002	−0.493	**
Rx peak (°)	180.52 ± 8.54	180.84 ± 24.02	181.96 ± 17.98	T1 vs. T2	0.671	0.289	ns
				T1 vs. T3	0.671	0.235	ns
				T2 vs. T3	0.727	−0.056	ns
Total range of Rx (°)	261.25 ± 92.44	328.46 ± 74.19	336.76 ± 57.45	T1 vs. T2	0.125	0.489	ns
				T1 vs. T3	0.125	0.530	ns
				T2 vs. T3	0.920	0.017	ns
Rz peak (°)	167.85 ± 24.25	142.09 ± 36.56	133.48 ± 48.18	T1 vs. T2	0.139	−0.556	ns
				T1 vs. T3	0.182	−0.461	ns
				T2 vs. T3	0.846	0.031	ns
Total range of Rz (°)	311.50 ± 54.55	246.80 ± 88.45	232.70 ± 111.32	T1 vs. T2	0.486	−0.400	ns
				T1 vs. T3	0.522	−0.287	ns
				T2 vs. T3	0.846	0.031	ns
Maximum Ty displacement (mm)	25.77 ± 9.53	27.22 ± 21.97	15.57 ± 9.51	T1 vs. T2	0.601	−0.156	ns
				T1 vs. T3	0.058	−0.600	ns
				T2 vs. T3	0.019	−0.425	*
Total range of Ty (mm)	28.24 ± 7.77	28.84 ± 22.30	18.74 ± 10.94	T1 vs. T2	0.548	−0.178	ns
				T1 vs. T3	0.092	−0.548	ns
				T2 vs. T3	0.092	−0.304	ns
Maximum longitudinal displacement, Tz (mm)	46.97 ± 13.13	51.09 ± 17.19	48.46 ± 14.67	T1 vs. T2	0.954	0.111	ns
				T1 vs. T3	0.954	−0.026	ns
				T2 vs. T3	0.954	−0.152	ns
Maximum Ry flexion angle (°)	62.51 ± 23.01	54.94 ± 17.60	50.68 ± 10.54	T1 vs. T2	0.426	−0.233	ns
				T1 vs. T3	0.426	−0.270	ns
				T2 vs. T3	0.426	−0.225	ns

Note: Data are presented as mean ± standard deviation. Pairwise comparisons were performed using the Brunner–Munzel test with Benjamini–Hochberg false discovery rate correction. RBC indicates rank–biserial correlation. Tx, lateral translation; Ty, anterior–posterior translation; Tz, proximal–distal translation; Rx, patellar tilt; Ry, patellar flexion; Rz, patellar axial rotation. Significance was defined by adjusted *p* values. *** *p* < 0.001; ** *p* < 0.01; * *p* < 0.05; ns, not significant.

**Table 4:** Comprehensive evaluation of dynamic kinematic features of patellar 6-DOF motion.

Degree of Freedom	Adjusted Significance	Effect Size	Time-Series Hierarchical Feature	Structural Role Determination
Tx	Yes	0.493–1.000	Continuous separation across full motion cycle	Dominant degree of freedom for phenotyping
Rx	No	0.530	Local synchronous fluctuation	Coordinated adjustment
Rz	No	0.556	Local phase-specific difference	Coordinated adjustment
Ty	Present locally	0.425	No stable gradient	Local difference
Tz	No	0.152	Highly overlapping curves	Stable
Ry	No	0.270	Highly overlapping curves	Stable

Note: Effect size is presented as the range of absolute RBC values for the corresponding degree of freedom. For Tx, the range was derived from both Tx peak and total range of Tx. Between-group comparisons were performed using the Brunner–Munzel test, with significance defined by BH-FDR-adjusted *p* values. Abbreviations: Tx = lateral translation; Ty = anterior–posterior translation; Tz = proximal–distal translation; Rx = patellar tilt; Ry = patellar flexion; Rz = patellar axial rotation.

## Data Availability

The raw data supporting the conclusions of this article will be made available by the authors upon request.

## Abbreviations

The following abbreviations are used in this manuscript:

4D-CT	Four-Dimensional Computed Tomography
6-DOF	6-degrees-of-freedom
BH-FDR	Benjamini–Hochberg false discovery rate
BO	Bisecting-offset index
IQR	Interquartile range
ns	Not significant
PFJ	Patellofemoral Joint
PMT	Patellar Motion Trajectory
RBC	Rank–biserial correlation
Rx	Patellar tilt
Ry	Patellar flexion
Rz	Patellar axial rotation
TT-TG	Tibial tuberosity–trochlear groove distance
Tx	Lateral translation
Ty	Anterior–posterior translation
Tz	Proximal–distal translation

## References

[1] Flandry F., Hommel G. (2011). Normal anatomy and biomechanics of the knee. Sports Med. Arthrosc. Rev..

[2] Mizuno Y., Kumagai M., Mattessich S.M., Elias J.J., Ramrattan N., Cosgarea A.J., Chao E.Y.S. (2001). Q-angle influences tibiofemoral and patellofemoral kinematics. J. Orthop. Res..

[3] Huberti H.H., Hayes W.C. (1984). Patellofemoral contact pressures. The influence of q-angle and tendofemoral contact. J. Bone Jt. Surg. Am..

[4] Dye S.F. (2005). The pathophysiology of patellofemoral pain: A tissue homeostasis perspective. Clin. Orthop. Relat. Res..

[5] Petersen W., Ellermann A., Gösele-Koppenburg A., Best R., Rembitzki I.V., Brüggemann G., Liebau C. (2014). Patellofemoral pain syndrome. Knee Surg. Sports Traumatol. Arthrosc..

[6] Duong V., Oo W.M., Ding C., Culvenor A.G., Hunter D.J. (2023). Evaluation and treatment of knee pain: A review. JAMA.

[7] Insall J., Salvati E. (1971). Patella position in the normal knee joint. Radiology.

[8] Merchant A.C., Mercer R.L., Jacobsen R.H., Cool C.R. (1974). Roentgenographic analysis of patellofemoral congruence. J. Bone Jt. Surg. Am..

[9] Laurin C.A., Lévesque H.P., Dussault R., Labelle H., Peides J.P. (1978). The abnormal lateral patellofemoral angle: A diagnostic roentgenographic sign of recurrent patellar subluxation. J. Bone Jt. Surg. Am..

[10] Zhan H., Zhao Z., Liang Q., Zheng J., Zhang L. (2025). Performance of artificial intelligence in automated measurement of patellofemoral joint parameters: A systematic review. J. Orthop. Surg. Res..

[11] Anvari A., Halpern E.F., Samir A.E. (2018). Essentials of statistical methods for assessing reliability and agreement in quantitative imaging. Acad. Radiol..

[12] Paiva M., Blønd L., Hölmich P., Barfod K.W. (2021). Effect of medialization of the trochlear groove and lateralization of the tibial tubercle on TT-TG distance: A cross-sectional study of dysplastic and nondysplastic knees. Am. J. Sports Med..

[13] Koh T.J., Grabiner M.D., De Swart R.J. (1992). In vivo tracking of the human patella. J. Biomech..

[14] Nagamine R., Otani T., White S.E., McCarthy D.S., Whiteside L.A. (1995). Patellar tracking measurement in the normal knee. J. Orthop. Res..

[15] van Kampen A., Huiskes R. (1990). The three-dimensional tracking pattern of the human patella. J. Orthop. Res..

[16] Katchburian M.V., Bull A.M., Shih Y.F., Heatley F.W., Amis A.A. (2003). Measurement of patellar tracking: Assessment and analysis of the literature. Clin. Orthop. Relat. Res..

[17] Adachi T., Kato Y., Kiyotomo D., Kawamukai K., Takazawa S., Suzuki T., Machida Y. (2023). Accuracy verification of four-dimensional CT analysis of knee joint movements: A pilot study using a knee joint model and motion-capture system. Cureus.

[18] Dandu N., Knapik D.M., Trasolini N.A., Zavras A.G., Yanke A.B. (2022). Future directions in patellofemoral imaging and 3D modeling. Curr. Rev. Musculoskelet. Med..

[19] Tran A., Lassalle L., Zille P., Guillin R., Pluot E., Adam C., Charachon M., Brat H., Wallaert M., D’aSsignies G. (2022). Deep learning to detect anterior cruciate ligament tear on knee MRI: Multi-continental external validation. Eur. Radiol..

[20] E T., Nai R., Liu X., Wang C., Liu J., Li S., Huang J., Yu J., Zhang Y., Liu W. (2023). Automatic measurement of the patellofemoral joint parameters in the Laurin view: A deep learning-based approach. Eur. Radiol..

[21] Wu Y., Chen P., Luo X., Huang H., Liao L., Yao Y., Wu M., Rangayyan R.M. (2016). Quantification of knee vibroarthrographic signal irregularity associated with patellofemoral joint cartilage pathology based on entropy and envelope amplitude measures. Comput. Methods Programs Biomed..

[22] Bahadır B., Sezgin E.A., Atik O.Ş. (2024). Established practices and future insights into patellar instability surgery: A review. Jt. Dis. Relat. Surg..

[23] Dejour H., Walch G., Nove-Josserand L., Guier C. (1994). Factors of patellar instability: An anatomic radiographic study. Knee Surg. Sports Traumatol. Arthrosc..

[24] Parikh S.N., Lykissas M.G., Gkiatas I. (2018). Predicting risk of recurrent patellar dislocation. Curr. Rev. Musculoskelet. Med..

[25] Vermeulen D., van der Valk M.R., Kaas L. (2019). Plaster, splint, brace, tape or functional mobilization after first-time patellar dislocation: What’s the evidence?. EFORT Open Rev..

[26] Pineda T., Dejour D.H. (2025). ‘À la carte’ treatment algorithm for patellofemoral instability. Rev. Esp. Cir. Ortop. Traumatol..

[27] Szybist S., Houser A., Corletto J., Maloy W. (2025). Patellofemoral biomechanics considerations: Analysis of factors contributing to patellofemoral pain. Curr. Sports Med. Rep..

[28] Neal B.S., Lack S.D., Bartholomew C., Morrissey D. (2024). Best practice guide for patellofemoral pain based on synthesis of a systematic review, the patient voice and expert clinical reasoning. Br. J. Sports Med..

[29] Heo J.W., Ro K.H., Lee D.H. (2019). Patellar redislocation rates and clinical outcomes after medial patellofemoral ligament reconstruction: Suture anchor versus double transpatellar tunnel fixation. Am. J. Sports Med..

[30] Garra S., Li Z.I., Carter T.R., Hamilton A.R., Pace J.L., Jazrawi L.M. (2024). Treatment of patellofemoral instability and chondral lesions. Instr. Course Lect..

[31] Fithian D.C., Paxton E.W., Stone M.L., Silva P., Davis D.K., Elias D.A., White L.M. (2004). Epidemiology and natural history of acute patellar dislocation. Am. J. Sports Med..

[32] Antinolfi P., Manfreda F., Placella G., Teodori J., Cerulli G., Caraffa A. (2018). The challenge of managing the “third-space” in total knee arthroplasty: Review of current concepts. Joints.

[33] Milinkovic D.D., Jovandic I., Zimmermann F., Balcarek P. (2022). The J-sign and the body mass index determine the disease-specific quality of life in patients with lateral patellar instability. Knee Surg. Sports Traumatol. Arthrosc..

[34] Dejour D.H., Mazy D., Pineda T., Cance N., Dan M.J., Giovannetti de Sanctis E. (2025). Patellar instability: Current approach. EFORT Open Rev..

[35] Xue Z., Song G.Y., Liu X., Zhang H., Wu G., Qian Y., Feng H. (2018). Excessive lateral patellar translation on axial computed tomography indicates positive patellar J sign. Knee Surg. Sports Traumatol. Arthrosc..

[36] Blønd L., Askenberger M., Stephen J., Akmeşe R., Balcarek P., El Attal R., Chouliaras V., Ferrua P., Monart J.M., Pagenstert G. (2025). Management of first-time patellar dislocation: The ESSKA 2024 formal consensus-Part 1. Knee Surg. Sports Traumatol. Arthrosc..

[37] Balcarek P., Blønd L., Beaufils P., Askenberger M., Stephen J.M., Akmeşe R., El Attal R., Chouliaras V., Ferrua P., Minguell Monart J. (2025). Management of first-time patellar dislocation: The ESSKA 2024 formal consensus-Part 2. Knee Surg. Sports Traumatol. Arthrosc..

[38] Misir A., Yuce A. (2025). AI in Orthopedic Research: A Comprehensive Review. J. Orthop. Res..

[39] Oettl F.C., Zsidai B., Oeding J.F., Hirschmann M.T., Feldt R., Fendrich D., Kraeutler M.J., Winkler P.W., Szaro P., Samuelsson K. (2025). Artificial Intelligence-Assisted Analysis of Musculoskeletal Imaging: A Narrative Review of the Current State of Machine Learning Models. Knee Surg. Sports Traumatol. Arthrosc..

[40] Eskandar K. (2026). The Role of Artificial Intelligence in Orthopedic Surgery: Current Applications and Future Perspectives—A Systematic Review of the Literature. Rev. Esp. Cir. Ortop. Traumatol..

[41] Pontoh L.A.P., Dilogo I.H., Fiolin J., Gosal S., Herdiman J.A., Hawali A.A., Kholinne E. (2026). Application of Artificial Intelligence in Orthopaedic Research: From Preclinical to Translational. J. Orthop. Surg..

